# Use of Kampo Diagnosis in Randomized Controlled Trials of Kampo Products in Japan: A Systematic Review

**DOI:** 10.1371/journal.pone.0104422

**Published:** 2014-08-13

**Authors:** Yoshiharu Motoo, Ichiro Arai, Kiichiro Tsutani

**Affiliations:** 1 Department of Medical Oncology, Kanazawa Medical University, Ishikawa, Japan; 2 Department of Kampo Medicine, Nihon Pharmaceutical University, Saitama, Japan; 3 Department of Drug Policy and Management, Graduate School of Pharmaceutical Sciences, The University of Tokyo, Tokyo, Japan; The National Institute for Health Innovation, New Zealand

## Abstract

**Background:**

The Committee for Evidence-based Medicine (EBM) of the Japan Society for Oriental Medicine started compiling Evidence Reports of Kampo Treatment (EKAT) in 2007. EKAT is a compilation of structured abstracts of randomized controlled trials (RCTs), along with comments by a third party reviewer. As of 31 December, 2012, there were 378 RCTs of Kampo medicines in Japan. The primary research question of this study is “How frequently is Kampo diagnosis used in RCTs of Kampo medicines?” The secondary research question is “When is Kampo diagnosis used in RCTs?”

**Materials and Methods:**

The structured abstract (SA) of each RCT article was reviewed to examine how Kampo diagnosis was used in RCTs, especially how Kampo diagnosis was used in the randomization process.

**Results:**

Kampo diagnosis was used before randomization in 27 RCTs (7.1%), after randomization in 31 RCTs (8.2%), and not used in 320 RCTs (84.7%). Before randomization, Kampo diagnosis was used as a criterion for inclusion in 10 RCTs, criterion for exclusion in 9 RCTs, and criteria for both inclusion and exclusion in 2 RCTs. Kampo formulas were determined according to Kampo diagnosis in 7 RCTs. After randomization, subgroup analyses according to Kampo diagnosis were done in 27 RCTs, and grade of disease severity at Kampo diagnosis was used for analysis as an endpoint in 4 RCTs.

**Conclusions:**

Kampo diagnosis was used before randomization only in approximately 15% of RCTs, and the number of RCT articles using Kampo diagnosis after randomization was almost the same as that before randomization. Further studies to determine the good RCTs conforming to CONSORT requirements and good systematic reviews conforming to PRISMA requirements are needed to clarify the significance of Kampo diagnosis.

## Introduction

Kampo, traditional Japanese medicine, originated from ancient Chinese medicine, and greatly developed especially during the Edo era (1603-186 A.D.). Kampo has distinct characteristics such as abdominal diagnosis for therapeutic indications and useful formulas with smaller amounts of herbs, compared with Chinese medicine. In 1967, four Kampo formulas were covered by National Health Insurance, and since 1986, 148 Kampo formulas have been approved for ethical use [1]. From 1967 to 1986, the ethical Kampo product approval process was based on “a consensus-based monograph” written by the Federation of Pharmaceutical Manufacturers' Associations of Japan under the supervision of the Ministry of Health and Welfare (re-named as the Ministry of Health, Labor and Welfare [MHLW] in 2001) and not on clinical evidence. The indications on the label were based on symptoms, not traditional Kampo medicine theory or Western medicine diagnosis. The MHLW reevaluation of 1985 to 2014 led to a slight modification of these indications.

In general, Kampo formulations are extracts of herbal formulas and prepared as described in the classical Kampo literature [2]–[4]. The difficulty with implementing randomized controlled trials (RCTs) of Kampo medicines has often been attributed to the use of Kampo diagnosis. Since high-quality GMP- based extracts of Kampo formulas for ethical use have been prescribed in RCTs, the reproducibility of RCT results have been very high. In Japan, Kampo is used in a Western-style medical system, and is prescribed by medical doctors, educated in Western medicine, but having basic knowledge of Kampo. Doctors are required, not by law, but as professionals, to have a basic knowledge of the indications for each Kampo formula at the time of prescription as well as knowledge of Western medical diagnosis. According to a survey by the Japan Kampo Medicines Manufacturers' Association (JKMA) in 2011, 52% of Japanese medical doctors prescribe Kampo formulas based on Western medicine, 32% on Western medicine with consideration of Kampo diagnosis, 10% on both Western medicine and Kampo medicine, and 6% on Kampo diagnosis [5].

Kampo diagnosis is based on the results of a physical examination, especially on the results of abdominal palpation, which is specific for Kampo medicine. Kampo medicine is taught at all 80 medical schools in Japan and is part of the Model Core Curriculum for Medical Education of 2001 set by the Association of Cooperative Researchers on Medical and Dental Education under the supervision of the Ministry of Education, Culture, Sports, Science and Technology of Japan. The Japan Society for Oriental Medicine (JSOM) has certified approximately 2,150 Kampo experts (medical doctors) as of March 2011. These experts have passed an examination and completed a training program. Indeed, ordinary physicians, not Kampo medicine experts, can prescribe Kampo drugs, and health insurance actually covers the cost of drugs prescribed for diseases as named in Western medicine name for the disease. Thus, Japanese doctors can prescribe Kampo formulas based on Western medical diagnosis. Since it is unclear that this system is adequate, evidence should be gathered from RCTs of Kampo medicines prescribed on the basis of Kampo diagnosis.

RCTs of Kampo medicines have been conducted in a variety of clinical fields such as gastroenterology, cardiology, respiratology, etc. The Committee for Evidence-based Medicine (EBM) of JSOM has investigated the description of Kampo products in the Japanese Clinical Practice Guidelines (CPGs), and presents the results of this investigation (in “Clinical Practice Guidelines Containing Kampo Products in Japan” [in Japanese]) to the public on the website (http://www.jsom.or.jp/medical/ebm/cpg/index.html). Analysis of the results indicates that CPG developers do not have sufficient access to the evidence on Kampo [3].

The first Evidence Reports of Kampo Treatment (EKAT) project started in 2001, and the first report was published in 2005. An Evidence Report Task Force (ER-TF) was established in 2005, and the EKAT project gathered momentum in 2007. The EKAT 2010 (which was published in 2010) contained the structured abstracts of 345 RCTs of Kampo formulas [6], [7], and has been linked to the Cochrane Library (CENTRAL) as a Specialized Register. EKAT 2013 will be available by July 2014 and covers all previous structured abstracts archived. However, not all the articles on Kampo RCTs include comments from a Kampo perspective. In addition, how Kampo diagnosis is used in each RCT has not been studied.

The primary research question of this study is “How frequently is Kampo diagnosis used in RCTs of Kampo medicines?” The secondary research question is “When is Kampo diagnosis used in RCTs?” The long-term goal of this research is to improve the quality of RCTs of Kampo treatment by showing the relevance of Kampo diagnosis to the evaluation of Kampo formulations.

## Materials and Methods

### 1. The process of compiling abstracts for EKAT 2010

Articles about RCTs of Kampo formulas (i.e., extract granules, tablets, and capsules, or pills, approved for prescription), published between 1986 and 2009, were included. References published before 1986 were excluded for the following reason: In 1986, the quality standard of Kampo products for ethical use was revised, and all the products needed to receive re-approval to meet the new standard. Therefore, all the Kampo products currently available on the market have the quality standards set in 1986. Kampo products meeting the old quality standard are no longer available, and information from clinical trials using these products is not applicable to current clinical practice. The data sources of EKAT were the Cochrane Library (CENTRAL), Igaku Chuo Zasshi (*Japana Centra Revuo Medicana* [JCRM], Ichushi) web, and the database offered by the Japan Kampo Medicines Manufacturers' Association (JKMA). The Cochrane Library (CENTRAL) includes PubMed/Medline-derived and EMBASE-derived RCTs. Studies on in-house formulas such as decoctions were excluded owing to their lesser reproducibility. Structured abstracts with comments by third parties were arranged in the order used in the International Classification of Diseases and Related Health Problems 10^th^ Revision (ICD10). Finally, 416 references were reviewed, and SAs of articles on 345 RCTs and 1 meta-analysis were prepared. Information on the development process in detail can be obtained on the website of EKAT 2010 [7]. EKAT has been updated every year.

### 2. Review of EKAT

**Figure 1:**
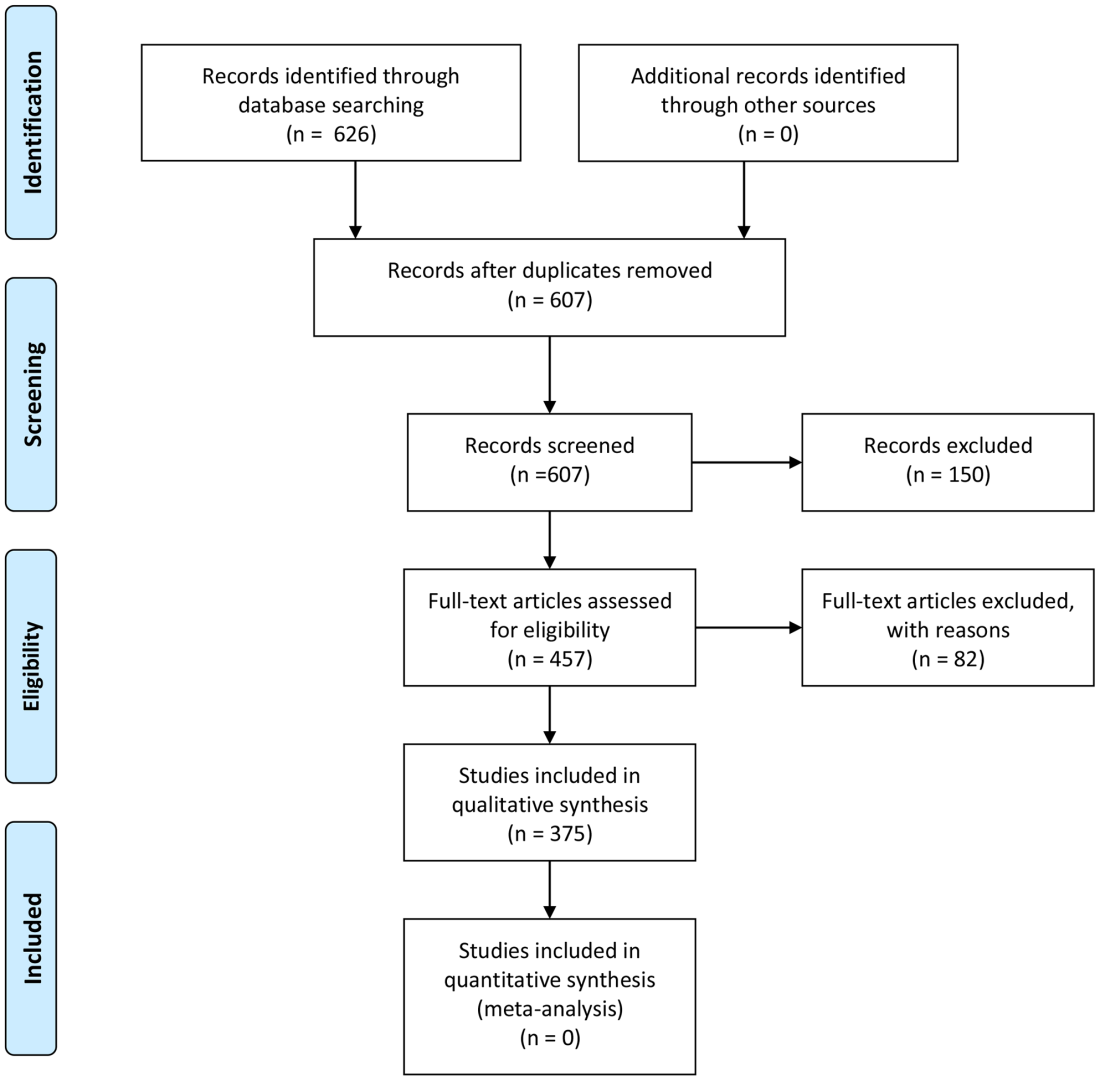
The PRISMA flow diagram.

We reviewed EKAT 2010 [7] and its supplementary versions EKAT Appendix 2011 [8] and EKAT Appendix 2012 [9]. Finally, 378 RCTs, 1 meta-analysis, and 457 articles were subjected to our analysis. Fig. 1 shows the PRISMA flow diagram [10]. In this diagram, the number of studies included in qualitative synthesis is 375, whereas the number of RCTs is 378. This difference was caused by the fact that 3 articles included 2 RCTs, respectively. We organized all the SAs in EKAT 2010, Appendix 2011, and Appendix 2012 into one package, classifying them into subspecialties such as cancer, infectious diseases, cardiovascular diseases, etc.

### 3. Identification of Kampo diagnosis in each SA

We reviewed each SA and its original article to determine whether Kampo diagnosis was used in the RCT process, focusing especially on the relationship between the application of Kampo diagnosis and randomization. Since each SA stated whether a Kampo diagnosis was made, the description of the Kampo diagnosis was easily retrievable. Each abstract in EKAT contains an item known as “From a Kampo medicine perspective”, which explains how Kampo diagnosis was used in the RCT.

### 4. The pre- and post-randomization uses of Kampo diagnosis

Each RCT was analyzed from the viewpoint of the pre-randomization and post-randomization uses of Kampo diagnosis. Kampo diagnosis was used at the design (pre-randomization) stage and data analysis (post-randomization) stage of RCTs. At the pre-randomization stage, Kampo diagnosis was used in the inclusion and/or exclusion criteria and description of Kampo diagnosis was included to make it understandable to prescribing physicians. At the post-randomization stage, subgroup analysis revealed a significant dependence of Kampo medicine effectiveness on Kampo diagnosis. We provided examples of how Kampo diagnosis was used at different stages of RCTs of Kampo treatments.

### 5. An example of a basic Kampo diagnosis

Kampo diagnosis of kidney deficiency requires the presence of 3 or more of the following 6 symptoms: 1) heaviness of the back; 2) heaviness in the lower legs with pain in the heels and lateral surface of the lower legs; 3) tinnitus/hearing loss; 4) loss of hair and hair luster; 5) looseness or loss of teeth; 6) sexual dysfunction (impotence, nocturnal emission).

## Results

SAs were prepared for 378 selected RCT references and 1 meta-analysis reference. The numbers of study designs in SAs are shown in Table 1.

**Table 1:** Designs of 378 RCTs in EKAT.

Randomization	
Randomized controlled trial (RCT)	348
Quasi-RCT/controlled clinical trial (CCT)	30
**Blinding**	
Double-blinded	36
Single-blinded	5
Open	337
**Assignment**	
Parallel	330
Crossover	48
Total	378

Use of Kampo diagnosis in 378 RCTs in EKAT 2010 (as well as appendices to EKAT 2010 [Appendix 2011 and 2012]) was analyzed. As shown in Table 2, Kampo diagnosis was used before randomization in 27 RCTs (7.1%) and after randomization in 31 RCTs (8.2%). There were 4 types of Kampo diagnosis use, i.e., usage in inclusion criteria, in exclusion criteria, in both inclusion and exclusion criteria, and in the selection of Kampo formulas. Kampo diagnosis was used for randomization of only responders in two RCTs and only non-responders in one RCT. Post-randomization subgroup analyses were performed in 27 RCTs, and Kampo diagnosis was used to determine the efficacy of Kampo formulations in 4 RCTs.

**Table 2:** Use of Kampo diagnosis in 378 RCTs in EKAT.

1. Pre-randomization	27 RCTs (7.1%)
1) Kampo diagnosis in inclusion criteria	10 (37.0%)
2) Kampo diagnosis in exclusion criteria	9 (33.3%)
3) Kampo diagnosis in inclusion criteria & exclusion criteria	2 (7.4%)
4) Selection of Kampo formula according to Kampo diagnosis	6 (22.2%)

2. Post-randomization	31 RCTs (8.2%)
1) Subgroup analyses according to Kampo diagnosis	27 (87.1%)
2) Severity score of Kampo diagnosis as endpoint	4 (12.9%)

Short descriptions including disease name, Kampo diagnosis, intervention (Kampo formula), and control are shown for each use category in Table 3. Further details can be found in each SA and its original article. Here, a typical RCT exemplifying a particular type of use is described below in narrative form.

**Table 3:** Short descriptions including disease name, Kampo diagnosis, intervention (Kampo formula), and control for each use category.

	Randomi zation	Disease	Kampo diagnosis	intervention	control	Author (year)
1	1 Inclusion criteria (n = 10)	Polycystic ovary syndrome	Sixty-four patients were randomly assigned to one of 2 groups using the diagnostic criteria “yin and yang, excess or deficiency, interior and exterior, cold and heat”, to receive 8-week preliminary administration of either keishibukuryogan or tokishakuyakusan. Then, 54 non-responder patients were further assigned to receive either a continuation of the same treatment (n = 27) or unkeito (n = 27) for 8 weeks.	Unkeito (n = 27)	Keishibukuryogan or Tokishakuyakusan (n = 27)	Ushiroyama T, et al. (2006)
2	1 Inclusion criteria (n = 10)	Sleep disorders	Of 20 normal healthy men receiving yokukansankachimpihange before the start of the study, 7 with sleep disorders favorably affected were selected for the study.	Yokukansankachimpi hange (n = 7)	Anchusan (n = 7)	Aizawa R, et al. (2002)
3	1 Inclusion criteria (n = 10)	Essential hypertension.	Excess pattern	Daisaikoto (n = 14)	No administration (n = 15)	Sasaki J, et al. (1993)
			Deficiency pattern	Chotosan (n = 24)	No administration (n = 30)	
4	1 Inclusion criteria (n = 10)	Common cold	Subject selection was made on the basis of persistent symptoms and discomfort in the mouth, which indicate “shosaikoto-sho”	Shosaikoto (n = 131)	Placebo (n = 119)	Kaji M, et al. (2001)
5	1 Inclusion criteria (n = 10)	Atopic dermatitis	*Qi*-deficiency was one of the inclusion criteria for enrollment in this trial.	Hochuekkito (n = 37)	Placebo (n = 40)	Kobayashi H, et al. (2010)
6	1 Inclusion criteria (n = 10)	Senile pruritus	intermediate pattern to excess pattern	Orengedokuto (n = 16)	Antihistamine (n = 16)	Ohkawara A, et al. (1991)
			deficiency pattern	Goshajinkigan (n = 25)	Antihistamine (n = 29)	
7	1 Inclusion criteria (n = 10)	Gonarthro sis	The *sho* concept was an inclusion criterion. Although “gonarthrosis complying with the *sho* for boiogitokabushi” was used as a criterion, the *sho* concept was not defined.	Boiogitoka shuchibushimatsu (n = 110)	Loxoprofen (n = 101)	Nishizawa Y, et al. (2007)
8	1 Inclusion criteria (n = 10)	Dysmeno rrhea	Forty females suffering from dysmenorrhea for at least 1 year, with all *qi* deficiency, *yin*, and static blood scores of 30 or more, without orthopedic disorders, and not receiving oral low-dose medications or prescribed anxiolytics.	Tokishakuyakusan (n = 20)	Placebo (n = 20)	Kotani N, et al. (1997)
9	1 Inclusion criteria (n = 10)	Climacteric complaints	Table 1, which shows the 7 target symptoms of climacteric disorder, was referred for selection of keishibukuryogan*.	Keishibukuryogan (n = 21)	Keishibukuryogan and tofisopam (n = 22)	Tanaka E, et al. (1997)
10	1 Inclusion criteria (n = 10)	Chronic headache	patients with chronic headache that responded to goshuyuto	Goshuyuto (n = 28)	Placebo(n = 25)	Odaguchi H, et al. (2005, 2006)
11	2 Exclusion criteria (n = 9)	Chronic hepatitis C	Patients with yin pattern and deficiency pattern were excluded before the allocation.	Shosaikoto (n = 49)	One of the commonly used liver protectors (n = 50)	Nakajima O et al. (1999)
12	2 Exclusion criteria (n = 9)	Interferon-resistant chronic hepatitis C	One patient with yin pattern and deficiency pattern was excluded before the allocation, and the study was actually conducted in 99 patients.	Shosaikoto (n = 39)	Squalene (n = 33) Cepharanthine (n = 40)	Nakajima O, et al. (2003)
13	2 Exclusion criteria (n = 9)	Spring nasal allergy (pollinosis)	Deficiency pattern patients were excluded.	Ryokankyomishingeninto (n = 15)	Shoseiryuto (n = 15)	Mori H, et al. (1996)
14	2 Exclusion criteria (n = 9)	Spring allergic rhinitis (pollinosis)	Since shoseiryuto is used in intermediate or excess pattern patients, and eppikajutsuto is used in physically strong patients, physically weak patients were excluded.	Eppikajutsuto (n = 49)	Shoseiryuto (n = 45)	Mori H, et al. (1997)
15	2 Exclusion criteria (n = 9)	Spring allergic rhinitis (pollinosis).	Since shoseiryuto is used in intermediate pattern to excess pattern patients, physically weak patients were excluded.	Daiseiryuto (Keishito + Makyokansekito) (n = 24)	Shoseiryuto (n = 45)	Mori H et al. (1998)
16	2 Exclusion criteria (n = 9)	Springtime nasal allergy and allergic conjuncti vitis	Patients with deficiency pattern were excluded*.	Keimakakuhanto (Keishito + Maoto) (n = 33)	Shoseiryuto (n = 32)	Mori, et al. (1999)
17	2 Exclusion criteria (n = 9)	Nasal allergy and allergic conjunctivi tis in spring	Subjects with deficiency pattern were excluded because shoseiryuto and gokoto are used to treat subjects with excess or intermediate patterns.	Gokoto (n = 58)	Shoseiryuto (n = 58)	Shimazaki Y, et al. (2001)
18	2 Exclusion criteria (n = 9)	Springtime nasal allergy and allergic conjunctivi tis	Patients with deficiency pattern were excluded*.	Maobushisaishinto (n = 32)	Shoseiryuto (n = 34)	Yoshimoto T, et al. (2002)
19	2 Exclusion criteria (n = 9)	Antibody production after influenza vaccination	Subjects not intending to use hochuekkito, as well as subjects with easy fatigability, a high susceptibility to colds, slow recovery from colds, a high susceptibility to other infections like herpes and wound infection, poor appetite, loose bowels, and somnolence especially after meals	Hochuekkito (n = 18)	Placebo (n = 18)	Hamazaki K, et al. (2007)
20	3 Inclusion & exclusion criteria (n = 2)	Hyper tension symptoms	The inclusion criteria were high blood pressure and presence of hypertension symptoms. Patients with cold/yin pattern or deficiency pattern were excluded. Patients with thin physique were also excluded.	Orengedokuto (n = 103)	Placebo (n = 101)	Arakawa K, et al. (2003, 2003)
21	3 Inclusion & exclusion criteria (n = 2)	Dyspepsia caused by dysfunction of the upper gastro intestinal tract	In this study, the inclusion criteria were deficiency pattern symptoms (i.e., decreased tone of abdominal wall, subjective/objective splashing sound, gastroptosis tendency, and mental/physical weakness) and the exclusion criteria were excess pattern symptoms (i.e., mental and physical strength, massive and muscular body, and reddish face).	Rikkunshito (n = 147)	low-dose (1:4 dilution) Rikkunshito (n = 133)	Harasawa S, et al. (1998, 1999)
22	4 Selection of Kampo formula according to Kampo criteria (n = 6)	Aqueous flare elevation after small-incision cataract surgery	Evaluation of *sho* and selection of Kampo formulations for each patient were conducted at the Kampo medicine clinic (now Department of Japanese Oriental Medicine) in the above-mentioned university hospital.	Orengedokuto (n = 14) Kakkonto (n = 10) Saireito (n = 10)	no medication (n = 20)	Ikeda N, et al. (2001)
23	4 Selection of Kampo formula according to Kampo criteria (n = 6)	Aqueous flare elevation after complicated cataract surgery	Evaluation of sho and selection of Kampo formulations for each patient were conducted at the Kampo medicine clinic(now Department of Japanese Oriental Medicine)in the above-mentioned university hospital.	Kakkonto (n = 12)	Saireito (n = 10)	Ikeda N, et al. (2002)
24	4 Selection of Kampo formula according to Kampo criteria (n = 6)	Common cold syndrome associated with fever	Kampo prescriptions were administered according to sho in patients with fever associated with common cold.	Kakkonto, Maoto, Keimakakuhanto, Chikujountanto, Shoseiryuto, Keishikashakuyakuto or Kososan (n = 35)	Fenoprofen (n = 45)	Homma Y, et al. (1995)
25	4 Selection of Kampo formula according to Kampo criteria (n = 6)	Peptic ulcer	Based on the endoscopic findings, patients with marked redness and irregularity of gastric antral mucosa were assigned to the shigyakusan treatment, and patients with less evident findings to the saikokeishito treatment.	Shigyakusan or Saikokeishito (n = 7)	Sucralfate (n = 6)	Watanabe H, et al. (1995)
26	4 Selection of Kampo formula according to Kampo criteria (n = 6)	Sjögren's syndrome	Arm 1 used Kampo diagnosis to allocate patients, specifically *jinkyo* (kidney deficiency). Kampo formulations for Arm 1 were selected based on the status of *jinkyo*: 1) bakumondoto alone for *jinkyo*-negative; 2) bakumondoto plus rokumigan for *jinkyo* without chills; and 3) bakumondoto plus hachimijiogan for *jinkyo* with chills.	Hochuekkito (n = 28)	Bakumondoto, Bakumondoto + Rokumigan or Bakumondoto + Hachimijiogan (n = 30)	Ohno S, et al. (2006)
27	4 Selection of Kampo formula according to Kampo criteria (n = 6)	Osteopenia in women during menopause	Keishibukuryogan for 6 patients with excess pattern, and tokishakuyakusan for 6 patients with deficiency pattern*.	Keishibukuryogan (n = 6) Tokishakuyakusan (n = 6)	no administration of Kampo drug (n = 6)	Ohta H, et al. (1990)

* described in the original article, not in SA.

### 1. Pre-randomization

#### 1. Kampo diagnosis in inclusion criteria

Kobayashi et al. [11] used Kampo diagnosis in their inclusion criteria. Their objective was to assess the efficacy of hochuekkito for the treatment of atopic dermatitis. Patients with atopic dermatitis and *qi* deficiency (n = 77) were included and divided into a hochuekkito administration group (n = 37: arm 1), and a placebo group (n = 40: arm 2). No significant between-arm difference was found in the reduction of skin lesion score and change of *qi* deficiency score.

These authors used response or non-response to hochuekkito as inclusion criteria, and randomized responders and non-responders as follows.

##### Allocation of responders to the Kampo formulation

Odaguchi et al. [12] evaluated the efficacy of goshuyuto for relief of chronic headache. Goshuyuto was administered for 4 weeks to 91 patients with chronic headache. Sixty patients responded to goshuyuto, 27 patients did not respond, and 4 patients withdrew. The 60 responders were randomly assigned either to arm 1 (goshuyuto, n = 28) or arm 2 (placebo, n = 25), and 53 patients completed this trial. The number of days with headache was significantly less in arm 1 than in arm 2. There was no significant between-arm difference in the dosage of analgesics taken.

##### Allocation of non-responders to the Kampo formulation

Ushiroyama et al. [13] reported the efficacy of switching to unkeito from treatment based on the traditional diagnostic criterion (i.e., “eight-principle pattern identification”) in women with polycystic ovary syndrome (PCOS). Among the 64 patients diagnosed with PCOS and treated with keishibukuryogan or tokishakuyakusan according to Kampo diagnosis, 54 non-responders were randomly assigned either to arm 1 (continuous administration of keishibukuryogan or tokishakuyakusan; n = 27) or arm 2 (unkeito; n = 27). Switching to unkeito decreased blood levels of luteinizing hormone and significantly stimulated ovulation. Unkeito had an ovulation-inducing action, regardless of Kampo diagnosis.

#### 2. Kampo diagnosis in exclusion criteria

Nakajima et al. [14] used Kampo diagnosis in their exclusion criteria. Their objectives were to assess the efficacy of shosaikoto for interferon-resistant chronic hepatitis C. There were 100 participants (i.e., patients with chronic active hepatitis C who completed interferon therapy) after exclusion of those with yin pattern and deficiency pattern and three assigned groups: squalene group (1500 mg/day, n = 33: arm 1), cepharanthine group (1 mg/kg body weight per day, n = 33: arm 2), and shosaikoto group (6.0 g/day, n = 34: arm 3). Efficacy was equivalent in all groups.

#### 3. Kampo diagnosis in inclusion and exclusion criteria

Arakawa et al. [15] evaluated the efficacy and safety of orengedokuto in patients with hypertension symptoms in a double-blind, randomized, controlled trial (DB-RCT). A total of 204 out of 265 patients met the inclusion criteria (presence of high blood pressure and hypertension symptoms such as irritability, etc., indicating the need for orengedokuto) and exclusion criteria, i.e., presence of yin pattern and deficiency pattern. They were divided into an orengedokuto administration group (n = 103: arm 1), and a placebo group (n = 101: arm 2). Hypertension symptoms were significantly lower in the orengedokuto group.

#### 4. Selection of Kampo formula according to Kampo diagnosis

Ohno [16] evaluated the efficacy of Kampo Medicine (as a system) in patients with Sjögren's syndrome. Sixty-four patients were assigned either to arm 1 (according to Kampo diagnosis, n = 30, after 2 dropped out) or arm 2 (n = 28; after 4 dropped out). Treatments included bakumondoto alone for kidney deficiency–negative (n = 23), bakumondoto plus rokumigan for kidney deficiency without coldness (n = 3), and bakumondoto plus hachimijiogan for kidney deficiency with coldness (n = 4) in arm 1 and hochuekkito in arm 2. The amount of increase in salivary secretions was significantly greater in arm 1 than in arm 2 (*P*<0.005).

### 2. Post-randomization

#### 1. Subgroup analyses according to Kampo diagnosis

Miyamoto et al. [17] reported the efficacy and safety of shoseiryuto in the treatment of bronchitis. Patients with mild to moderate bronchitis and evaluable symptoms were randomly assigned to two arms (arm 1: shoseiryuto, n = 101; arm 2: placebo, n = 91). General improvement was moderate to marked and tended to be greater in arm 1 than in arm 2. Subgroup analyses revealed that patients without physical frailty and those with cough and watery sputum showed a significantly higher rate of general improvement in arm 1 than in arm 2.

#### 2. Severity score of Kampo diagnosis as endpoint

Shimada [18] evaluated the efficacy and safety of tokishakuyakusan for the treatment of hypofunction and decreased independence in patients with post-stroke. Such patients were randomly assigned to two arms (arm 1: tokishakuyakusan, n = 16; arm 2: no administration of Kampo medicines, n = 15). There were statistical differences between the two arms in the Stroke Impairment Assessment Set and Functional Independence Measure scores at 12 months. These scores remained at baseline levels in arm 1 but were significantly decreased in arm 2. By additional exploratory analysis using severity scores of Kampo diagnosis such as blood stasis and kidney deficiency as endpoints in before and after comparison, the authors found a significant improvement in blood stasis score and kidney deficiency score in arm 1 at 12 months. But, blood stasis score did not change and kidney deficiency score worsened in arm 2, resulting in significant differences between the two arms at 12 months.

## Discussion

In this study, we described the current status of the use of Kampo diagnosis in RCTs of Kampo formulations. In EKAT, two Japanese-language databases (Ichushi [JCRM] and JKMA) and one English-language database (Cochrane Library CENTRAL) were searched. Addition of Chinese-language databases to the conventional systematic review (SR) increased the number of references retrieved by the search [19]. However, the quality of the RCTs of Kampo medicines is most important, and our SR revealed that EKAT provides us usable information on Kampo treatment. Our report is the first paper to divide the uses of Kampo diagnosis into pre- and post-randomization uses, to describe the four pre-randomization uses of Kampo diagnosis (in inclusion criteria, exclusion criteria, both inclusion and exclusion criteria, and in Kampo formula selection), and to describe the two post-randomization uses of this diagnosis (subgrouping of patients for analysis, use of a score for Kampo diagnosis severity as an endpoint).

Most RCTs of Kampo medicines were small and did not use Kampo diagnosis. This might imply that Kampo medicines show efficacy in the Western-style medical system and this information would be useful to clinicians whose practice is oriented toward Western medicine. To show the relevance of Kampo diagnosis to the conduct of RCTs on Kampo treatment, the quality and quantity of RCTs should be improved. If we could verify the relevance of the use of Kampo diagnosis to such RCTs, it is expected that their findings would be more significant and that Kampo medicine efficacy and safety would be increased. It is vitally important for traditional East Asian medicine to address this issue.

Clinical research on Kampo treatments, especially by researchers of Western medicine, is often criticized by Kampo traditionalists for not using Kampo diagnosis in the randomization process. However, no report has shown how Kampo diagnosis is used in RCTs. Most criticisms of RCTs that ignore Kampo diagnosis come from individuals who have poor understanding of modern evaluation methodology. The use of traditional diagnosis in RCTs or CPGs has been discussed [20], [21]. The present study, however, does not discuss the superiority of Kampo-diagnosis use over its non-use. Kampo-diagnosis use and non-use were compared in only 4 RCTs (which is the fourth type of pre-randomization usage designated “Selection of Kampo formula according to Kampo diagnosis”. When the number of RCTs using Kampo diagnosis for this purpose increases to a certain level, the research question “Is the use of Kampo diagnosis superior to its non-use?” can be answered by performing another SR. Preliminary analysis has revealed the poor quality of articles reporting RCTs. To show the superiority of Kampo diagnosis use with SRs, it will be necessary to improve the quality of these studies by using the CONSORT statement [22].

There are pros and cons to the use of Kampo diagnosis in RCTs. If the Kampo diagnosis is found to enhance the efficacy of Kampo treatment, it would broaden the applications of Kampo medicine in Western disease entities. If the Kampo diagnosis is not found to enhance the efficacy of a Kampo formula, it would mean that Kampo products can have favorable effects without the need to know complex concepts and techniques of Kampo diagnosis. However, the prescription of a Kampo formula based on Kampo diagnosis could increase the drug's safety and thereby lead to better clinical outcome.

It is too early to know the precise implications or interpretation of legal policy or the labeling of ethical Kampo products. More RCTs of drugs prescribed according to Kampo diagnosis are needed to compare with RCTs of drugs not prescribed according to Kampo diagnosis and show the superiority in both efficacy and safety of Kampo formulations. The current labeling policy in Japan is believed to be sufficient, based on the fact that Western medical diagnosis was used in over 90% of RCTs in our study. While Kampo traditionalists stress the importance of using the Kampo diagnostic system, they lack interest in regulatory systems and emphasize educational systems.

As for the limitations of the SR, firstly, there was possible publication bias. Clinical trial registration (the tool to prevent publication bias) was developed in 2005 in Japan [23]. The number of articles about the RCTs in this study is as follows: 1986-89: 11 (2.9%), 1990s: 180 (47.6%), 2000s: 157 (41.5%), 2010-11s:30 (7.9%). There were 261 RCT articles from 1986 to 2004, and 117 RCT articles from 2005 to 2011. Thus, 69.0% of RCTs were published before the problem of publication bias was addressed. Secondly, the motivations for conducting RCTs were not clearly stated; some were of purely academic interest, and some were of industrial interest. The CONSORT statements of 1996, 2001, and 2010 have been translated into Japanese but were ignored by those interested in Kampo medicine. And only the statement of 2010 addresses the role of the funding agency in the conduct of the study. Kampo industry may have more interest in RCTs that involve the use of Western medicine diagnosis because reporting them increases sales. PRISMA guidelines [24] should be used when preparing systematic reviews of RCTs with low risk of biased information.

## Conclusions

Kampo diagnosis before randomization was used only in approximately 15% of RCTs, and the number of RCT articles using Kampo diagnosis after randomization was almost the same as that before randomization. To clarify the importance of Kampo diagnosis in the conduct of RCTs, the CONSORT statement should be used in the reporting of good RCTs and the PRISMA statement should be used in the reporting of systematic reviews.

## Supporting Information

Checklist S1 **PRISMA Checklist**
(DOC)
